# Exciton Dissociation in a Model Organic Interface: Excitonic State-Based Surface Hopping versus Multiconfigurational Time-Dependent Hartree

**DOI:** 10.1021/acs.jpclett.2c01928

**Published:** 2022-07-28

**Authors:** Wei-Tao Peng, Dominik Brey, Samuele Giannini, David Dell’Angelo, Irene Burghardt, Jochen Blumberger

**Affiliations:** † Department of Physics and Astronomy and Thomas Young Centre, University College London, London WC1E 6BT, United Kingdom; ‡ Institute of Physical and Theoretical Chemistry, Goethe University Frankfurt, Max-von-Laue-Strasse 7, 60438 Frankfurt am Main, Germany

## Abstract

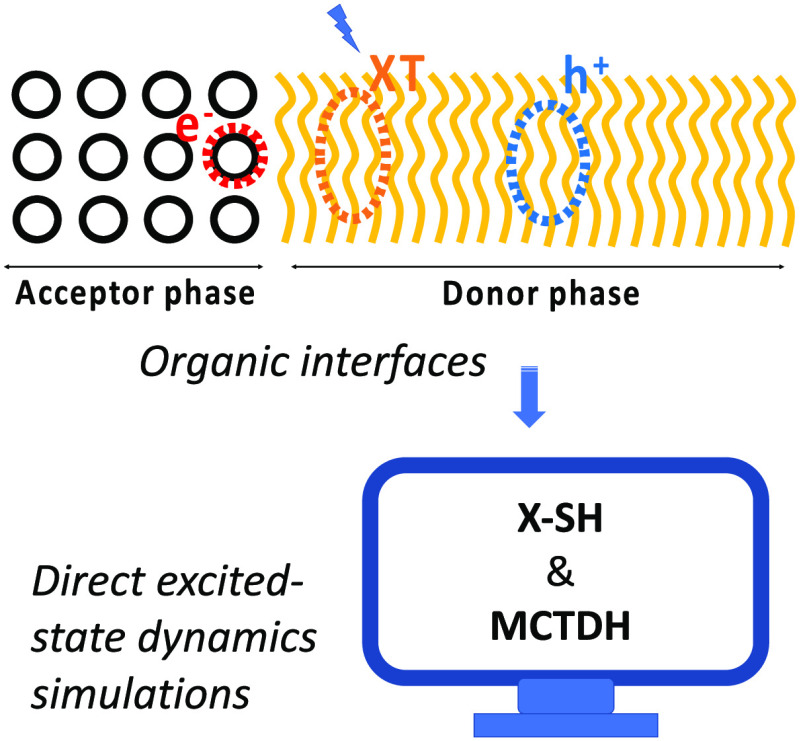

Quantum dynamical simulations are essential for a molecular-level understanding of light-induced processes in optoelectronic materials, but they tend to be computationally demanding. We introduce an efficient mixed quantum-classical nonadiabatic molecular dynamics method termed eXcitonic state-based Surface Hopping (X-SH), which propagates the electronic Schrödinger equation in the space of local excitonic and charge-transfer electronic states, coupled to the thermal motion of the nuclear degrees of freedom. The method is applied to exciton decay in a 1D model of a fullerene–oligothiophene junction, and the results are compared to the ones from a fully quantum dynamical treatment at the level of the Multilayer Multiconfigurational Time-Dependent Hartree (ML-MCTDH) approach. Both methods predict that charge-separated states are formed on the 10–100 fs time scale via multiple “hot-exciton dissociation” pathways. The results demonstrate that X-SH is a promising tool advancing the simulation of photoexcited processes from the molecular to the true nanomaterials scale.

Organic solar cells (OSCs) have attracted much research interest over the last few decades because of a number of beneficial features, such as low manufacturing cost, mechanical flexibility, light weight, and environmentally friendly materials. One of the major parameters determining the performance of a solar cell is the power conversion efficiency (PCE), and the best OSC designs to date have reached over 19%.^1−3^ To further optimize OSC materials and devices, an improved understanding of the fundamental optoelectronic processes is essential.

Various spectroscopy approaches have been applied to elucidate the charge separation mechanisms in OSCs.^4−11^ However, because of the complexity of such systems, contradictory results have been reported in the literature. One prominent example is the role of the lowest-lying charge transfer (CT_1_) state, namely, the bound electron–hole state at the donor–acceptor interface. Though many studies suggest it is the gateway state for charge separation independently of the time scale of exciton dissociation,^4−8^ other works claim that the charge separation is mainly through ultrafast hot carrier dissociation instead of the CT_1_ dissociation.^9−11^ It has been suggested that these discrepancies are primarily because of differences in the interfacial energetics, which are in turn because of the different molecular constituents, compositions, and diverse fabrication processes adopted that cause distinct morphology for donor and acceptor domains,^12,13^ which subsequently alter the parameters that govern the light-induced processes in the OPVs.

Theoretical modeling, however, can be carried out for structurally well-defined and increasingly realistic model systems. This allows us, in principle, to investigate how the mechanism of charge generation and separation depends on structural and energetic parameters.^14−23^ Common theoretical models often describe the excited-state processes in OSC materials in terms of rate theories that have been developed for molecular donor–acceptor systems (e.g., Marcus–Levich–Jortner). When applied to materials, they typically come with a number of restrictive assumptions. For instance, they neglect spatial delocalization of excitons and charge carriers, which is expected to have a profound effect on exciton dissociation and charge transport efficiencies.

Direct quantum dynamical simulation of the coupled electron–nuclear motions that account for such phenomena would be highly desirable, but they tend to be computationally expensive when applied to materials.^11,24−29^ In this regard, important progress has been made by carrying out Multiconfiguration Time-Dependent Hartree (MCTDH) and related Multilayer MCTDH (ML-MCTDH) calculations,^30,31^ for the quantum dynamics of nonadiabatic nuclear motion on vibronic coupling models parametrized to excited-state electronic structure calculations.^32^ A variety of complex processes in model systems could be treated in this way, e.g., exciton diffusion in an oligothiophene chain,^33^ electron–hole separation in donor–acceptor conjugated co-oligomers,^34^ and charge separation in the donor–acceptor junctions.^35,36^

Widely considered the gold standard for quantum dynamics, MCTDH is computationally expensive. Notwithstanding the impressive progress that has been recently made to increase the number of vibrational modes that can be treated in ML-MCTDH (several hundreds),^33−36^ applications to truly nanoscale systems (>10 nm) that require several hundreds if not thousands of molecules to be simulated at quantum level is likely to be out of reach for some time. Computationally more efficient approximate methods, e.g., mixed quantum-classical nonadiabatic molecular dynamics (MQC-NAMD) such as decoherence-corrected trajectory surface hopping,^37^ are a viable alternative for large systems.^38−42^ Some of us have recently developed surface hopping methodologies (termed Fragment Orbital-Based Surface Hopping (FOB-SH^43−45^) and Frenkel Exciton Surface Hopping (FE-SH^46^)) for the quantum dynamical propagation of charge carriers or Frenkel excitons in nanoscale materials, e.g., organic crystals^46−48^ and (disordered) thin films.^49^ Experimental charge mobilities and exciton diffusion constants could be well reproduced for a range of materials, and the simulations revealed a clear correlation between the spatial delocalization of the charge carrier or exciton and the respective diffusion constants.

In this work, we have implemented an extension of our previously developed FOB-SH and FE-SH methods by combining and coupling the electronic-state spaces for charge transfer and localized (Frenkel) exciton states. This enables us to simulate exciton transport coupled to exciton dissociation, charge generation and charge separation at the level of trajectory surface hopping, which was not possible with either FOB-SH or FE-SH. We dub our extension eXcitonic state-based Surface Hopping (X-SH) to emphasize the applicability of the method to light-induced excited-state processes in molecular materials. For simplicity, we consider a 1D chain composed of *N* electron donating chromophore molecules and one effective electron-accepting molecule, as shown in Figure 1a, and only nearest-neighbor couplings. Generalization to larger systems in 2D or 3D and to couplings beyond nearest neighbors is straightforward. In X-SH, the total Hamiltonian is constructed as
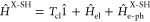
1where *T*_cl_ is the classical kinetic energy of the nuclei,  is the electronic Hamiltonian, and  is the electron–phonon coupling term.  can be divided into XT and CS blocks and a XT–CS off-diagonal block,
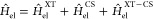
2where
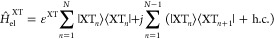
3
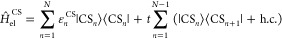
4and
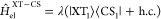
5

**Figure 1:**
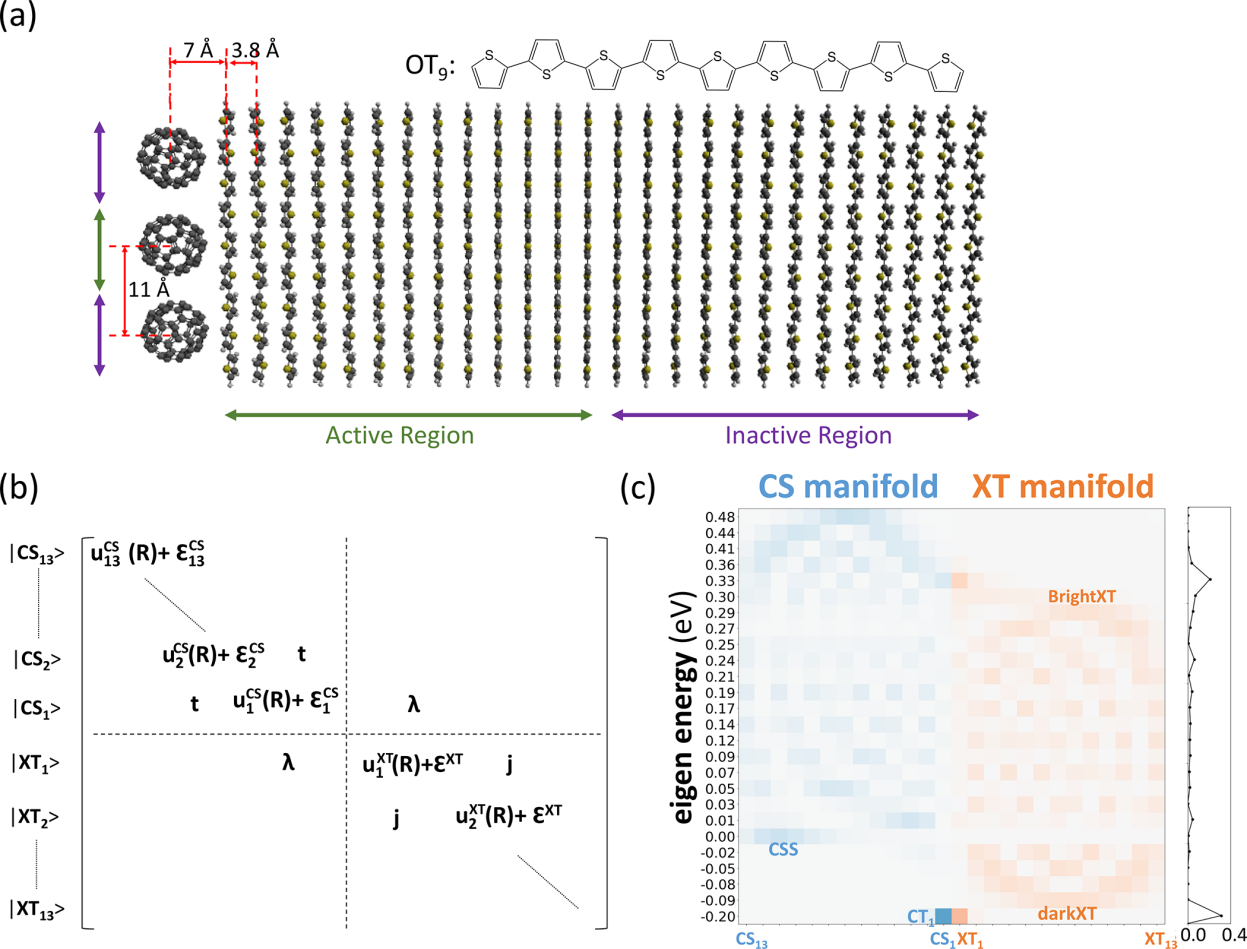
(a) Model system for the fullerene–oligothiophene single junction. (b) Electronic Hamiltonian matrix in the basis of diabatic Frenkel exciton (XT) and charge-separated states (CS). See the main text for definition of the matrix elements. (c) Energies and diabatic-state populations of the adiabatic electronic states at the electronic ground-state minimum geometry (absolute values squared of the eigenvectors, in orange for XT and in blue for CS diabatic states). The sidebar to the right depicts the projection of the diabatic XT_1_ state, used as the initial state for X-SH and MCTDH simulations, onto the adiabatic eigenstates.

In eq 3, ε^XT^ is the electronic energy offset between diabatic XT_*n*_ and CS_1_ states, where ε^XT^ = *E*_XT_*n*__ – *E*_CS_1__ in the minimum energy configuration of the ground state (**R**_GS_). ε_*n*_^CS^ is the electronic energy of diabatic states CS_*n*_, where donor molecule *n* carries a positive charge and the acceptor molecule a negative charge at configuration **R**_GS_. The local electron–phonon part is written as
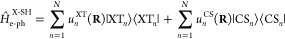
6

The term *u*_*n*_^XT/CS^(**R**) is the electronic energy of state *n* at a nuclear configuration R, relative to the energy of that state at **R**_GS_. Thus, *E*_*n*_^XT^(**R**) = ε^XT^ + *u*_*n*_^XT^(**R**) is the electronic energy of state XT_*n*_ at nuclear configuration **R**, i.e., the total potential energy of the system when site *n* is in the excited state and all other sites are in the (neutral) ground electronic state. Similarly, *E*_*n*_^CS^(**R**) = ε_*n*_^CS^ + *u*_*n*_^CS^(**R**) is the energy of state CS_*n*_ at nuclear configuration **R**, i.e., the total potential energy of the system when the acceptor molecule is negatively charged and the *n*th donor molecule is positively charged while all other molecules are in the neutral ground electronic state. Here, all electronic and excitonic couplings are fixed; i.e., nonlocal electron–phonon coupling is not included. Moreover, energetically accessible excited states other than the ones considered, e.g., charge transfer excitonic states within the donor manifold, triplet states, etc., are not considered but could, in principle, be included by extension of the electronic-state space.

The time-dependent wave function is expressed as a linear combination of the XT and CS states
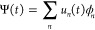
7where  and , *n* = 1, *N*, and *u*_*n*_ is the corresponding expansion coefficient. The wave function is propagated according to the time-dependent Schrödinger equation,
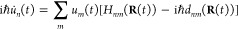
8where  are the elements in the electronic Hamiltonian described above. The nonadiabatic coupling elements (NACE) between the quasi-diabatic states, , are typically very small and are neglected. We have shown in our previous study on charge transport that neglecting the NACE gives essentially the same dynamics but accelerates the calculations considerably.^48^ The nuclei are propaged on a single adiabatic potential energy surface (the active state) at every time step and are allowed to hop stochastically between different surfaces according to Tully’s fewest switching hopping probablility.^37^ The Heisenberg decoherence time for the exponential damping of the coefficients of all except the active electronic states is applied to correct the overcoherence problem in the surface hopping method.^50,51^ A state tracking algorithm is used to detect trivial crossings between the adiabatic states.^44^

Here we apply X-SH to simulate the exciton decay dynamics in an one-dimensional (1D) model of a fullerene–oligothiophene interface, which was previously investigated at the level of MCTDH.^35,36^ We also report new ML-MCTDH computations significantly extending the simulation time from previously 1 to 10 ps. Both approaches employ the same electronic Hamiltonian (see eqs 2–5 and equation (S1)), which enables us to compare X-SH with MCTDH dynamics on an equal footing for a complex, application-relevant system. This allows us to assess the trade-offs that the two approaches make: classical treatment but inclusion of all nuclear degrees of freedom (DoF) in X-SH versus full quantum mechanical treatment of a limited number of DoF in MCTDH. In particular, it affords an assessment of the effect of classical vs quantum treatment of nuclear motion on the exciton relaxation mechanism and dynamics. A certain caveat is that the MCTDH calculations, which include zero-point energy, were restricted to *T* = 0 K whereas X-SH calculations were carried out at a classical temperature of *T* = 300 K. In this respect, it is worth noting that the classical nuclei at 300 K may actually lead to a narrower distribution of initial conditions in the phase space than that obtained from sampling a Wigner distribution for the ground vibrational state.^52^

The atomistic model of the oligothiophene (OT_9_)–fullerene (C_60_) donor–acceptor interface is shown in Figure 1a. In X-SH, the system is composed of 3 C_60_ molecules and 26 OT_9_ molecules with periodic boundary conditions along the direction of the stack. However, as in MCTDH, only one C_60_ unit and 13 OT_9_ molecules closest to that fullerene are treated as electronically active. The molecules in the inactive region maintain the structural integrity of the electronically active region during X-SH molecular dynamics, thus effectively replacing the bulk environment. Consequently, 13 (quasi-)diabatic Frenkel excitonic states in the donor phase are considered (eq 3). The state where the Frenkel exciton (XT) is located on the *n*th OT_9_ molecule is labeled XT*_n_*, *n* = 1, ..., 13. Similarly, the charge-separated (CS) state (eq 4) with the hole on the *n*th OT_9_ molecule and the electron on the C_60_ molecule is labeled CS*_n_*. The corresponding electronic Hamiltonian matrix for the 26 electronic states is shown in Figure 1b. The site energies (diagonal elements) for the XT and CS states were calculated with a fully atomistic force field in X-SH (eq 6 and Supporting Information equations (S6)–(S11)), and with a linear vibronic coupling potential along eight effective modes for each molecule in MCTDH^27,37^ (Supporting Information equations (S3)–(S5)). The atomistic force field for XT and CS states was parametrized to reproduce exactly the total reorganization energy for exciton transfer and for charge transfer used in MCTDH; see the Supporting Information for details. Furthermore, in both approaches, XT and CS_1_ states are offset by 0.1 eV at the Franck–Condon point (Δ*E*_offset_ = ε^XT^ – ε_1_^CS^ = 0.1 eV), and the Coulomb energy of the CS states was modeled by an effective Coulomb barrier (i.e., “Coulomb barrier 1” of ref (36) representing an OT_13_-(C_60_)_7_ aggregate; shown in Figure S1 and labeled as ε_*i*_^CS^ in Figure 1b for site *i*). The coupling (or off-diagonal) elements in the electronic Hamiltonian between nearest neighbors were fixed to the values used in ref (35); all other coupling matrix elements were set to zero. For XT–XT couplings, *j* = 0.1 eV, for CS–CS couplings, *t* = −0.12 eV, and for the XT–CS coupling at the interface, λ = −0.2 eV (note the sign for the latter was incorrectly reported in ref (35)). Δ*E*_offset_, the Coulomb barrier, and coupling values are based on previously reported time-dependent density functional theory (TDDFT) and DFT calculations and a diabatization scheme using adiabatic states from TDDFT as basis set.^53^ They are reproduced in the SI for convenience.

Prior to reporting the results of the nonadiabatic dynamics simulations, we analyze the electronic eigenstates (or adiabatic electronic states) of our model interface as obtained by diagonalization of the electronic Hamiltonian at the electronic ground-state minimum geometry. In Figure 1c, the eigenstates are projected on the diabatic (site) basis of XT (orange) and CS states (blue). The lowest-lying eigenstate in the system is the interfacial charge transfer state, which we label CT_1_ hereafter. It is mostly composed of the CS_1_ (54%) and XT_1_ (31%) diabatic states. The energy of this state, −0.20 eV, is lower than those for CS_1_ (0 eV) and XT_1_ (0.1 eV) because of the electronic coupling between these two states, λ = −0.2 eV and, to a lesser extent, between CS_1_ and CS_2_ states. (A simple two-state model including CS_1_ and XT_1_ states only would give an energy *E*(CT_1_) = ^1^/_2_(Δ*E*_offset_ – (Δ*E*_offset_^2^ + λ^2^)^0.5^) = −0.16 eV.) The XT manifold of eigenstates is centered at Δ*E*_offset_ = 0.1 eV, and the bandwidth is ∼4*j* = 0.4 eV, as expected from a tight binding model. Thus, the lower edge of the XT band is at −0.09 eV, 0.11 eV above CT_1_. It is mainly composed of XT_5_–XT_9_ states and denoted as the dark XT state because an H-aggregate of OT_9_ molecules (1D packing with positive XT–XT couplings) is considered, where the lowest state is optically inaccessible.^35^ The upper edge at 0.29 eV is formed of the bright XT state, which is mainly composed of XT_6_–XT_9_. Finally, the CS manifold of eigenstates is centered at 0.24 eV, which is slightly below the maximum of the Coulomb barrier, and the bandwidth is ∼4*t* = 0.48 eV. The lower CS band edge is formed by a charge-separated state (CSS) composed of diabatic CS states that are furthest away from the interface, CS_8_–CS_13_. The energy of this state, 0.00 eV, defines the binding energy of the interfacial charge transfer state CT_1_, 0.20 eV.

We have carried out nonadiabatic dynamics simulations choosing the fully localized XT_1_ diabatic state as initial state and report the results in Figure 2. X-SH and MCTDH predict a similar decay dynamics of the XT_1_ state at short times (<200 fs, Figure 2a); within 100 fs the populations of the dark XT state and the CSS accumulate markedly, to 0.13 (0.17) and 0.11 (0.11) at 100 fs, respectively, for X-SH (MCTDH). Beyond 100 fs, some differences are clearly discernible. In MCTDH, the population of the dark XT state keeps increasing, up to 0.25 at 1 ps, whereas in X-SH, it decays after 100 fs by converting to the CT_1_ state (as indicated by the diabatic CS_1_ population in Figure 2). The population difference between the dark XT state from the two approaches is very close to the difference in the CT_1_-state population (as indicated by two arrows of similar length in Figure 2b). At longer times, >1 ps, the populations for the dark XT and CSS states decay in MCTDH, but significant populations remain at 10 ps (Figure 2c and Figure S1). At this point, the quantum equilibrium population of electronic states (from imaginary time propagation; see Figure S4) has not been reached. By contrast, in X-SH, both dark XT and CSS have fully decayed after a few picoseconds. This establishes the classical equilibrium population for all electronic states in the system to a good approximation (dash-dotted lines in Figure 2c; see the Supporting Information for their calculation), showing that the newly developed X-SH method satisfies the detailed balance in the long-time limit, similarly to FOB-SH^50^ and FE-SH.^46^

**Figure 2:**
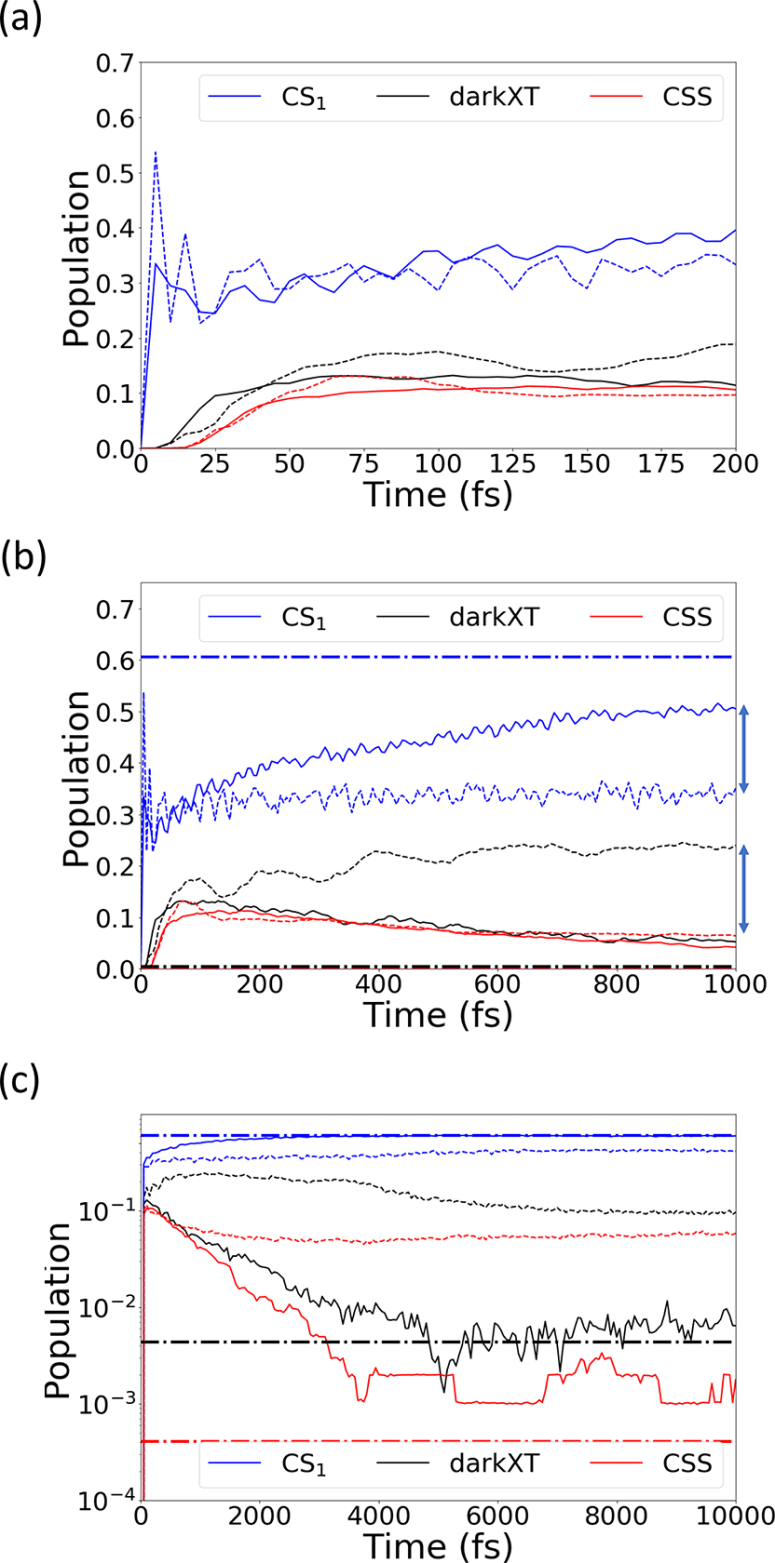
Population dynamics of the interfacial state (CS_1_), the charge-separated state (CSS), and the dark XT state (dark XT) from X-SH (solid lines) and MCTDH (dashed lines) simulations, initialized from the XT_1_ diabatic state. (a)–(c) show the dynamics up to 200 fs, 1 ps, and 10 ps, respectively. The horizontal lines (dash-dotted) in (b) and (c) indicate the thermal equilibrium (Boltzmann) populations of the electronic states at 300 K. (c) is reproduced in linear scale in Figure S2. The nuclear and electronic time steps are 0.1 and 0.02 fs, respectively. The convergence of the populations with respect to time step can be found in Figure S3.

The fast decay dynamics in X-SH after 100 fs could be an artifact of the classical treatment of nuclear motion. Similar observations have been reported by Freixas et al., who found that (decoherence-corrected) surface hopping exhibited a faster deexcitation dynamics than their reference method, multiconfigurational Ehrenfest with ab initio multiple cloning (whereas Ehrenfest, unsurprisingly, gave a too slow decay).^54^ However, the decay dynamics in MCTDH could be somewhat underestimated because of the zero-temperature conditions and the limited number of modes included in the model. During radiationless decay of XT_1_ to the CT_1_ state, up to 0.5 eV of electronic energy needs to dissipate via the nuclear degrees of freedom. Because only eight intramolecular modes per molecule (112 in total) are included, in the absence of intermolecular modes, the excess electronic energy cannot efficiently dissipate. This is likely to result in a slight overestimation of excited electronic states (dark XT, CSS) and prevents full relaxation to the equilibrium populations. In X-SH, all nuclear DoF, i.e., all intra- and intermolecular modes, are included (5604 in total), in particular the low-frequency modes that, as we show below, act as a heat bath absorbing the excess electronic energy and establishing thermal equilibrium.

In the following, we trace the energy flow to the nuclear vibrations during the electronic relaxation process in X-SH employing a normal-mode analysis (NMA), similarly as in ref (55). To this end, the total kinetic energy is projected on the 189 intramolecular normal modes of each OT_9_ molecule in the electronically active region, further details are given in the Supporting Information. The kinetic energy of each mode averaged over all X-SH trajectories is shown in Figure 3 as a function of time. Most of the modes fluctuate stably around the mean thermal energy, *k*_B_*T*/2 = 13 meV at 300 K, at any time (Figure 3b). These modes do not couple with the electronic transitions and can be interpreted as spectator or bath modes during the de-excitation process. Only six intramolecular high-frequency modes (depicted in Figure S5) exhibit significant excess kinetic energy during the first ∼500 fs of electronic relaxation; see Figure 3a. This is followed by dissipation of the excess kinetic energy to the spectator modes. After a few picoseconds, the vibrational energy redistribution is essentially complete. Thus, our analysis of the X-SH dynamics agrees with the assumption made in MCTDH simulations that only a few high-frequency modes are important in the relaxation process.^35,36^ In fact, the six intramolecular modes identified in Figure 3a have frequencies (734–3228 cm^–1^) that are similar to those of the six highest frequency modes selected in MCTDH (902–3238 cm^–1^). However, our results also highlight the importance of including a sufficient number of bath modes to reach the equilibrium distribution.

**Figure 3:**
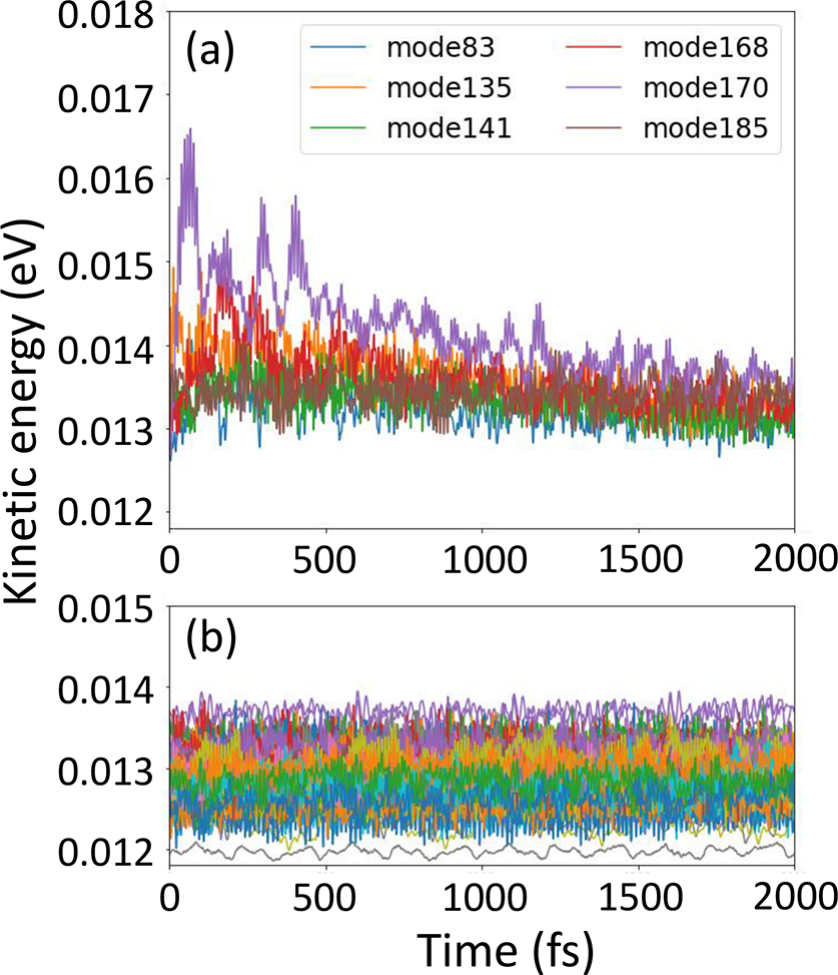
Dissipation of electronic excitation energy by vibrational modes in X-SH simulations. Normal modes with kinetic energy exceeding 14 meV in at least one instance within the first 2 ps of relaxation dynamics are shown in (a) and denoted “active” modes. The kinetic energy fluctuations of all other normal modes, denoted “spectator” modes, are shown in (b). The six “active” modes are indicated in Figure S5. The kinetic energy of each mode was averaged over 1000 X-SH trajectories; the thermal average, *k*_B_*T*/2, is 13 meV at 300 K.

Analyzing 1000 X-SH trajectories, we identify seven distinct exciton decay pathways to the energetically lowest CT_1_ state, which we briefly summarize in the following (see also Table 1 with percentage of occurrence given and Figure 4 for representative X-SH trajectories). (1) As the initial XT_1_ diabatic state projects to 34% on the CT_1_ adiabat (see the sidebar in Figure 1c), it can convert directly to CT_1_ through decoherence. XT_1_ is also observed to relax to CT_1_ (2) via the XT manifold, Figure 4a, (3) by the CS manifold without formation of the CSS state, Figure 4b, or (4) by first passing through the XT and then through the CT manifold or (5) vice versa or (6) via XT–CT hybrid states. Finally, (7) XT_1_ can form the charge-separated state (CSS) by passing through the CS manifold, Figure 4c.

**Figure 4:**
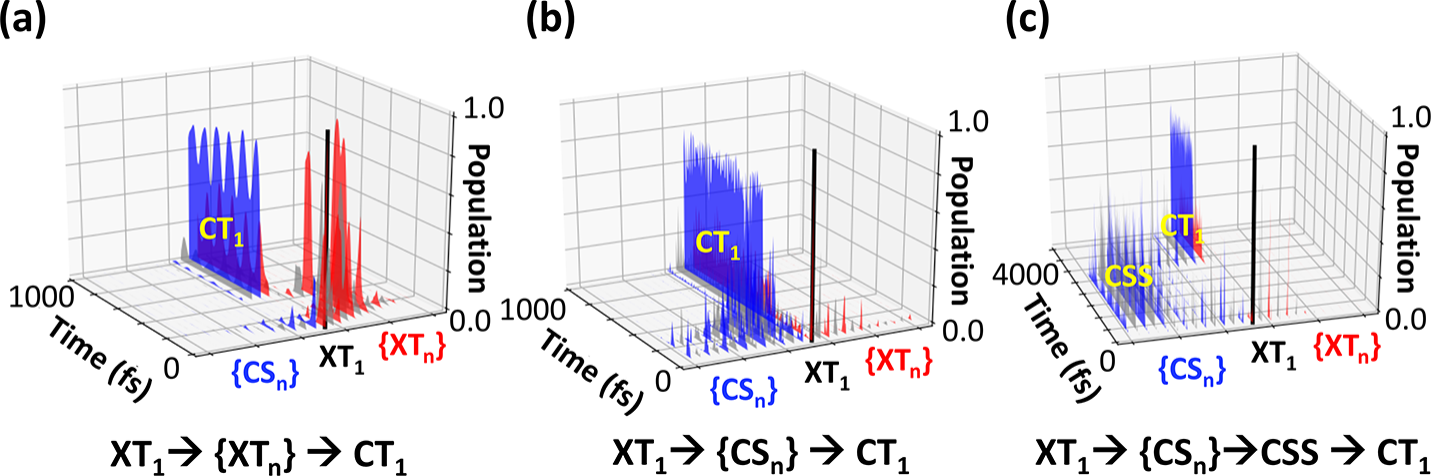
Three pathways for exciton dissociation as obtained from X-SH: (a) XT_1_ → {XT*_n_*} → CT_1_, (b) XT_1_ → {CS*_n_*} → CT_1_, and (c) XT_1_ → {CS*_n_*} → CSS → CT_1_. Initially, all population is on the XT_1_ state (black bar). The XT manifold {XT*_n_*} is color-coded alternately in red and gray, and the CS manifold {CS*_n_*} in blue and gray. XT_1_ to XT_13_ are arranged from center to right, and CS_1_ to CS_13_ populations are arranged from center to left. The pathways in (a), (b), and (c) are denoted as pathways (2), (3), and (7) in Table 1.

**Table 1:** Seven Pathways for Exciton Relaxation from the XT_1_ State, as Obtained from X-SH Simulation^a^

	pathways	percentage
(1)	XT_1_ → CT_1_	33.7
(2)	XT_1_ → {XT*_n_*} → CT_1_	24.7
(3)	XT_1_ → {CS*_n_*} → CT_1_	6.3
(4)	XT_1_ → {XT*_n_*} → {CS*_n_*} → CT_1_	0.5
(5)	XT_1_ → {CS*_n_*} → {XT*_n_*} → CT_1_	3.8
(6)	XT_1_ → {XT*_n_*, CS*_n_*} → CT_1_	17.0
(7)	XT_1_ → {CS*_n_*} → CSS → CT_1_	14.0

a The percentage gives the number of trajectories within the ensemble of 1000 X-SH trajectories following the pathway indicated. A trajectory is classified as reaching the {XT*_n_*}, {CS*_n_*}, or CSS manifold when the sum of XT_1_ to XT_13_, CS_1_ to CS_13_, or CS_8_ to CS_13_ populations is >95%, >95%, or >80%, respectively, in at least one instance in time.

The formation of the desired CSS state via pathway (7) may be best described as “hot carrier dissociation”. However, because of the finite system size of our model, virtually all of the generated CSS population converts to CT_1_ within a few picoseconds. Because the charge carriers in the CSS state are beyond the maximum of the Coulomb barrier, in a larger model, they would become free carriers, swept toward the electrodes by the electric field or recombining bimolecularly at longer times (geminate recombination). Once the system has relaxed to CT_1_ via pathways (1)–(7), the escape from that state is negligible (<1%) on the time scale of present simulations (10 ps), unsurprisingly because CT_1_ is >0.2 eV below the next highest states, CSS and the dark XT state in our model. Thus, for the given model parameters employed, our simulations predict that charge carrier generation occurs exclusively via the “hot carrier dissociation” pathway on the 10 ps time scale of present simulations. Though this does not exclude the possibility for dissociation of CT_1_ to the CSS state (“cold carrier dissociation” pathway), which may well occur on longer, nanosecond time scales and other material parameters.

In summary, we have extended our surface hopping methodology to enable the simulation of exciton dissociation to charge carriers in molecular materials. We found that the developed X-SH approach agrees very well with ML-MCTDH in predicting the decay dynamics and state populations at ultrafast time scales (<100–200 fs) as well as the intramolecular modes to which the excess energy is transferred. Thereafter, X-SH predicts a fairly rapid decay to the equilibrium populations of electronic states in a few picoseconds, not seen in MCTDH, possibly because of the lack of low-frequency bath modes or missing temperature effects in the latter calculations. MCTDH calculations incorporating those effects might clarify this issue in the future. On the whole, nuclear quantum effects such as nuclear tunneling and zero point motion that are missing in X-SH, do not seem to be overly important for the prediction of the population dynamics, at least for the current model at room temperature. This is likely because of the strong electronic coupling between the states in our model facilitating relaxation to the ground state without having to cross high barriers where tunneling and zero point motion are likely to become important. Thus, we conclude that X-SH appears to be a promising method for the simulation of exciton relaxation processes in this regime that is characteristic for organic optoelectronic materials.

A major advantage of X-SH compared to fully quantum dynamical methods is its computational efficiency permitting the simulation of realistic 2D and possibly 3D nanoscale systems (>10 nm) comprising hundreds or more molecular sites. Moreover, it is straightforward to include in a realistic manner many of the effects that have not been included in the present study: charge delocalization in acceptor and donor, thermal fluctuations of excitonic and electronic couplings (i.e., off-diagonal electron–phonon couplings) in donor and acceptor phases and at the interface, recombination to the electronic ground state, influence of interface geometry and static disorder. Work in our laboratory is currently ongoing to include these effects in X-SH.

## Supporting Information Available

Additional details of X-SH and MCTDH methods, the analysis of thermal equilibrium populations and the energy redistribution in the X-SH approach, a table of reorganization energies, and figures of Coulomb barriers and each site, population dynamics, convergence of time-dependent populations, population of electronic states, and vibrational modes (PDF)
