# Supplementary material for: Exciton Dissociation in a Model Organic Interface: Excitonic State-Based Surface Hopping versus Multiconfigurational Time-Dependent Hartree
Source: J Phys Chem Lett. 2022 Jul 28;13(31):7105–12. doi: 10.1021/acs.jpclett.2c01928 (PMC9376959; doi:10.1021/acs.jpclett.2c01928)
Supplement: Supplementary file 1 — jz2c01928_si_001.pdf [file jz2c01928_si_001.pdf]

Supporting Information

Exciton Dissociation in a Model Organic Interface: Excitonic State-based Surface Hopping versus Multi-Configurational Time-Dependent Hartree

Wei-Tao Peng[1], Dominik Brey[2], Samuele Giannini[1,3], David Dell'Angelo[1], Irene Burghardt[2], Jochen Blumberger[1]**

[1]Department of Physics and Astronomy and Thomas Young Centre, University College London, London WC1E 6BT, United Kingdom

[2]Institute of Physical and Theoretical Chemistry, Goethe University Frankfurt, Max-von-Laue-Str. 7, 60438 Frankfurt/Main, Germany

[3]Present address: Laboratory for Chemistry of Novel Materials, University of Mons, Mons 7000, Belgium

AUTHOR INFORMATION

Corresponding Author

I. Burghardt

Email: burghardt@chemie.uni-frankfurt.de

J. Blumberger

Email: j.blumberger@ucl.ac.uk

Electronic Hamiltonian

In this study, we adopt the same 1D model of a fullerene-oligothiophene junction as used in Reference 1. In MCTDH, the total electron-nuclear Hamiltonian is composed of the kinetic ($\hat{T}\hat{1}$), the electronic ($\hat{H}_{el}$) and the local electron-phonon ($\hat{H}_{e\text{-}ph}^{MCTDH}$) terms,

$$\hat{H}^{MCTDH} = \hat{T}\hat{1} + \hat{H}_{el} + \hat{H}_{e\text{-}ph}^{MCTDH}. \quad \text{(S1)}$$

In this study, both MCTDH and X-SH employ the same electronic Hamiltonian $\hat{H}_{el}$. Details of $\hat{H}_{el}$ can be found in the main text (equations (2)-(5)).

MCTDH and X-SH differ in the treatment of the nuclear degrees of freedom and thus in the definition of the kinetic and local electron-phonon ($\hat{H}_{e\text{-}ph}$) parts.[1] In MCTDH,

$$\hat{T} = \sum_{l=1}^{N_F} \frac{\omega_l^F}{2} (\hat{p}_l^F)^2 + \sum_{n=1}^{N} \sum_{l=1}^{N_{OT}} \frac{\omega_{n,l}^{OT}}{2} (\hat{p}_{n,l}^{OT})^2 \quad \text{(S2)}$$

is the nuclear kinetic energy operator in mass- and frequency-weighted coordinates. Further, $N = 13$ refers to the number of OT fragments, and $N_F = N_{OT} = 8$ effective modes are considered per fragment.

The electron-phonon (vibronic) coupling part of the MCTDH Hamiltonian is given in terms of Linear Vibronic Coupling (LVC) terms for the fullerene and thiophene parts:

$$\hat{H}_{e\text{-}ph}^{MCTDH} = \hat{H}_{e\text{-}ph}^{F} + \hat{H}_{e\text{-}ph}^{OT} \quad \text{(S3)}$$

where the Hamiltonian reads as follows in mass and frequency weighted coordinates,

(i) for N_F fullerene super-particle modes,

$$\hat{H}_{e-ph}^{F} = \sum_{l=1}^{N_F} \left(\frac{\omega_l^F}{2} (\hat{q}_l^F)^2 + \sum_{n=1}^{N} c_l^F \hat{q}_l^F \left| CS_n \right\rangle \left\langle CS_n \right| \right) \quad \text{(S4)}$$

(ii) for $N \times N_{OT}$ oligothiophene modes,

$$\hat{H}_{e-ph}^{OT} = \sum_{n=1}^{N} \sum_{l=1}^{N_{OT}} \left(\frac{\omega_{n,l}^{OT}}{2} (\hat{q}_{n,l}^{OT})^2 + c_{CS,l}^{OT} \hat{q}_{n,l}^{OT} \left| CS_n \right\rangle \left\langle CS_n \right| + c_{XT,l}^{OT} \hat{q}_{n,l}^{OT} \left| XT_n \right\rangle \left\langle XT_n \right| \right) \quad \text{(S5)}$$

Here, it is assumed that the OT electron-phonon couplings are identical for all fragments (such that the vibronic couplings $c_{CS,l}^{OT}$ and $c_{XT,l}^{OT}$ do not carry a site index n)

Local electron-phonon coupling in X-SH

The energy of the neutral electronic ground state is calculated using the standard Generalized Amber Force Field (GAFF).[2] The energy of state XT_n is calculated using suitably modified force field parameters for the excited state of the nth OT9 molecule and the standard GAFF force field parameters for the other molecules. The force field parameter for the excited state are based on the standard GAFF force field. Only the equilibrium bond lengths were changed so as to reproduce the reorganization energy obtained from DFT ground state and TDDFT excited state calculations on an isolated OT9 molecule in the gas phase (at CAM-B3LYP/6-31G(d,p)[3] level). The resultant reorganization energy for excitation of a single OT9 molecules is

$$\lambda_{XT}^{OT} = \frac{1}{2}\left(\left[E_{XT}^{OT}(R_{GS}) + E_{GS}^{OT}(R_{XT})\right] - \left[E_{XT}^{OT}(R_{XT}) + E_{GS}^{OT}(R_{GS})\right]\right) \tag{S6}$$

where $E_{XT}^{OT}(R_{XT})$ and $E_{XT}^{OT}(R_{GS})$ are the excited state energies and $E_{GS}^{OT}(R_{XT})$ and $E_{GS}^{OT}(R_{GS})$ the ground state energies of the isolated OT9 molecules in the minimum energy configuration of the excited state (R_{XT}) and ground state (R_{GS}), respectively. The reorganization energy obtained , 0.254 eV, from DFT/TDDFT was somewhat larger than the value used in MCTDH calculations in Ref 1, 0.137 eV, probably because not all modes are included in the latter. To facilitate comparison between MCTDH and X-SH, the bond displacements were scaled by a factor of 0.73 to match the reorganization energy Eq. S6 from the force field with the value used in MCTDH, the latter defined in terms of linear electron-phonon couplings ($c_{XT,i}$) and the vibrational frequencies ($\omega_{XT,i}$) of the effective modes considered,

$$\lambda_{XT}^{OT} = \sum_i \frac{c_{XT,i}^2}{2\hbar\omega_{XT,i}} \tag{S7}$$

A similar approach was adopted to calculate the energy of state CS_n. The force field was suitably modified to describe the C_{60} anion and the positively charged state of the nth OT9 molecule, and for all other molecules the default GAFF parameters were taken. Again, the force field parameters for the C_{60}^- and $OT9^+$ are based on the standard GAFF force field. Only the equilibrium bond lengths were changed so as to reproduce the reorganization energy obtained from DFT calculation for the neutral and charged isolated molecules in the gas phase (at B3LYP/6-31G(d,p) level[4-6]), resulting in reorganization energies of 0.070 and 0.101 eV for C_{60} and OT9,

$$\lambda^{F} = \frac{1}{2}\left(\left[E_{CS}^{F}(R_N) + E_{N}^{F}(R_{CS})\right] - \left[E_{CS}^{F}(R_{CS}) + E_{N}^{F}(R_N)\right]\right) \tag{S8}$$

$$\lambda_{CS}^{OT} = \frac{1}{2}\left(\left[E_{CS}^{OT}(R_N) + E_{N}^{OT}(R_{CS})\right] - \left[E_{CS}^{OT}(R_{CS}) + E_{N}^{OT}(R_N)\right]\right) \tag{S9}$$

The DFT bond displacements were scaled by factors of 0.99 and 0.82 to match the reorganization energies used in MCTDH, 0.069 and 0.068 eV.

$$\lambda^F = \sum_i \frac{(c_i^F)^2}{2\hbar\omega_i^F} \tag{S10}$$

$$\lambda_{CS}^{OT} = \sum_i \frac{(c_{CS,i}^{OT})^2}{2\hbar\omega_{CS,i}^{OT}} \tag{S11}$$

The reorganization energy for charge separation (in the limit of infinite separation of OT and F) is $\lambda_{CS} = \lambda^F + \lambda_{CS}^{OT}$ = 0.137 eV. We summarize all the reorganization energies and the values used in Reference 1 in Table S1.

We note that electrostatic interactions are not included in the force field description. The effective Coulombic interaction between the hole and the electron in the CS states is already included in the $\varepsilon_{CT}(n)$ term. Van-der Waals interactions are modelled by standard Lennard-Jones terms and remain unchanged for the different electronic states.

Nuclear forces in X-SH

The nuclear forces on the active potential energy surface E_a are expressed as

$$F_a = -\nabla_R E_a = -\left[\mathbb{U}^\dagger\left(\nabla_R \mathbb{H}\right)\mathbb{U}\right]_{aa}, \tag{S12}$$

where the $\mathbb{H}$ is the matrix representation of $\hat{H} = \hat{H}_{el} + \hat{H}_{e\text{-}ph}^{X\text{-}SH}$, and $\mathbb{U}$ the unitary matrix diagonalizing $\mathbb{H}$.[7] The nuclear gradient of the Hamiltonian matrix contains $\nabla_R H_{nn}$ and $\nabla_R H_{nn'}$ terms. As described above, the H_{nn} elements (the diagonal terms in the Hamiltonian matrix) in the X-SH approach are composed of local electron-phonon couplings $u(R)$ evaluated from classical force field and the applied constant energy ε^{XT} and ε_n^{CS} terms at XT and CS states. As a result, the $\nabla_R H_{nn}$ can be evaluated from classical force field and the contributions from the gradients of the constant energy shift are zero. In addition, because all off-diagonal elements $H_{nn'}$ of the Hamiltonian matrix are constant (frozen couplings), $\nabla_R H_{nn'} = 0$.

Thermal equilibrium populations in the X-SH approach

For the thermal equilibrium populations, we perform the simulations with a thousand trajectories on the lowest-lying state in the model system with Born-Oppenheimer approximation (without hops between states) and propagate each trajectory for 2 ps. The

equilibrium population P_i for each diabatic state i is calculated using following equations at T = 300 K,

$$P_i = \left\langle \sum_a w_a \times P_{ai} \right\rangle , \tag{S13}$$

where

$$w_a = \frac{\exp[-\Delta E_a / k_B T]}{\sum_b \exp[-\Delta E_b / k_B T]} . \tag{S14}$$

The w_a is the weight of an adiabatic state a at each time step, where ΔE_a is the energy difference between state a and the lowest-lying state (the state the Born-Oppenheimer MD is running on) in the system. The P_{ai} is the contribution of diabatic state i in the adiabatic state a and $\langle . \rangle$ represents the time and trajectory average. As a result, P_i gives the equilibrium population of the diabatic state i which corresponds to one of the twenty-six (XT1~XT13 and CS1~CS13) states. In the main text Figure 2c, we plot the equilibrium populations of the CS1, the darkXT (XT5~XT9) and the CSS (CS8~CS13) states.

The Energy Redistribution from the Normal Mode Analysis in the X-SH Approach

In the normal mode analysis, we adopt the procedures from literature.[8] In short, the mass-weighted Hessian matrix of OT9 molecule

$$\mathbb{H}_{ij}(\boldsymbol{R}_N) = -\partial^2 E / \partial q_i \partial q_j \tag{S15}$$

is diagonalized to obtain the eigenvectors with elements l_{ji}. In the equation, q_i is the mass weighted Cartesian coordinate, and R_N is the equilibrium geometry of the neutral ground state. The l_{ji} and R_N are obtained at B3LYP/6-31G(d,p) level of theory. The momentum of a vibrational mode i can be obtained by

$$\dot{Q}_i(t) = \sum_{j=1}^{3N} l_{ji} \dot{q}_j(t) . \tag{S16}$$

where $\dot{q}_j(t)$ is the velocity (in mass-weighted Cartesian coordinate) at time t that is from the molecular trajectory of our X-SH simulations. As a result, the projected kinetic energy on the i-th vibrational mode at every time step is

$$K_i(t) = \frac{1}{2}\dot{Q}_i^{\,2}, \tag{S17}$$

where $i = 1 \sim 3N - 6$.

Table S1. Reorganization energies λ (in eV) obtained from present DFT calculations. For X-SH simulations presented in this work, the DFT values were scaled to the ones used in MCTDH.

	**DFT**	**MCTDH**
λ_{XT}^{OT}	0.254	0.137
λ_{CS}^{OT}	0.101	0.068
λ^{F}	0.070	0.069
$\lambda_{XT->CS}^{OT}$	0.110	0.057

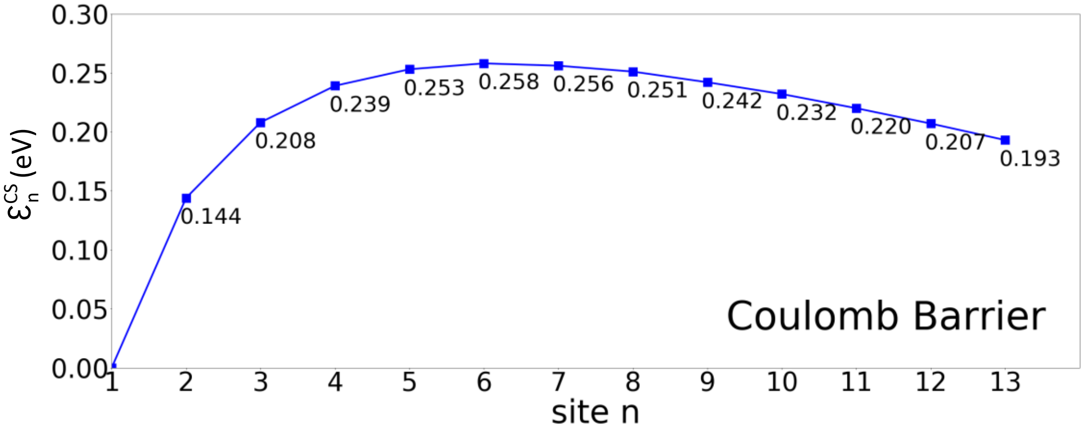

Figure S1. The Coulomb barrier ε_n^{CS} at each site n applied in this study.

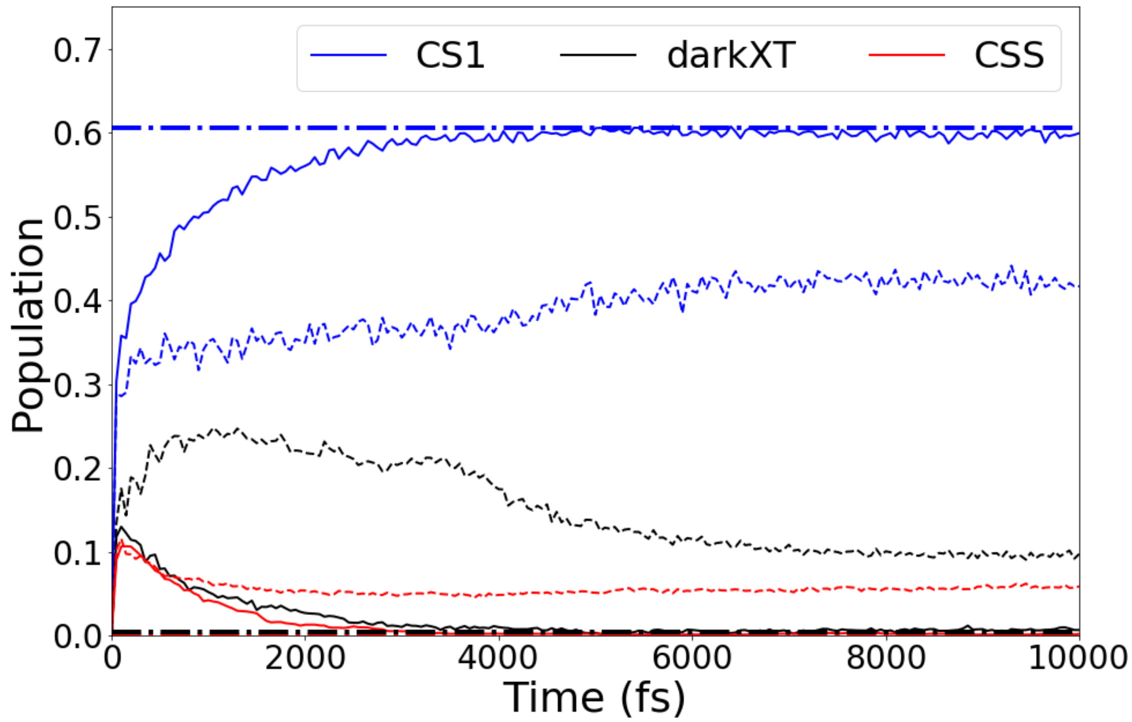

Figure S2. The population dynamics of the MCTDH (dashed lines) and X-SH (solid lines) approaches for the time scale up to 10 ps. The horizontal lines (dash-dotted) represent the thermal equilibrium populations at 300K.

(a)

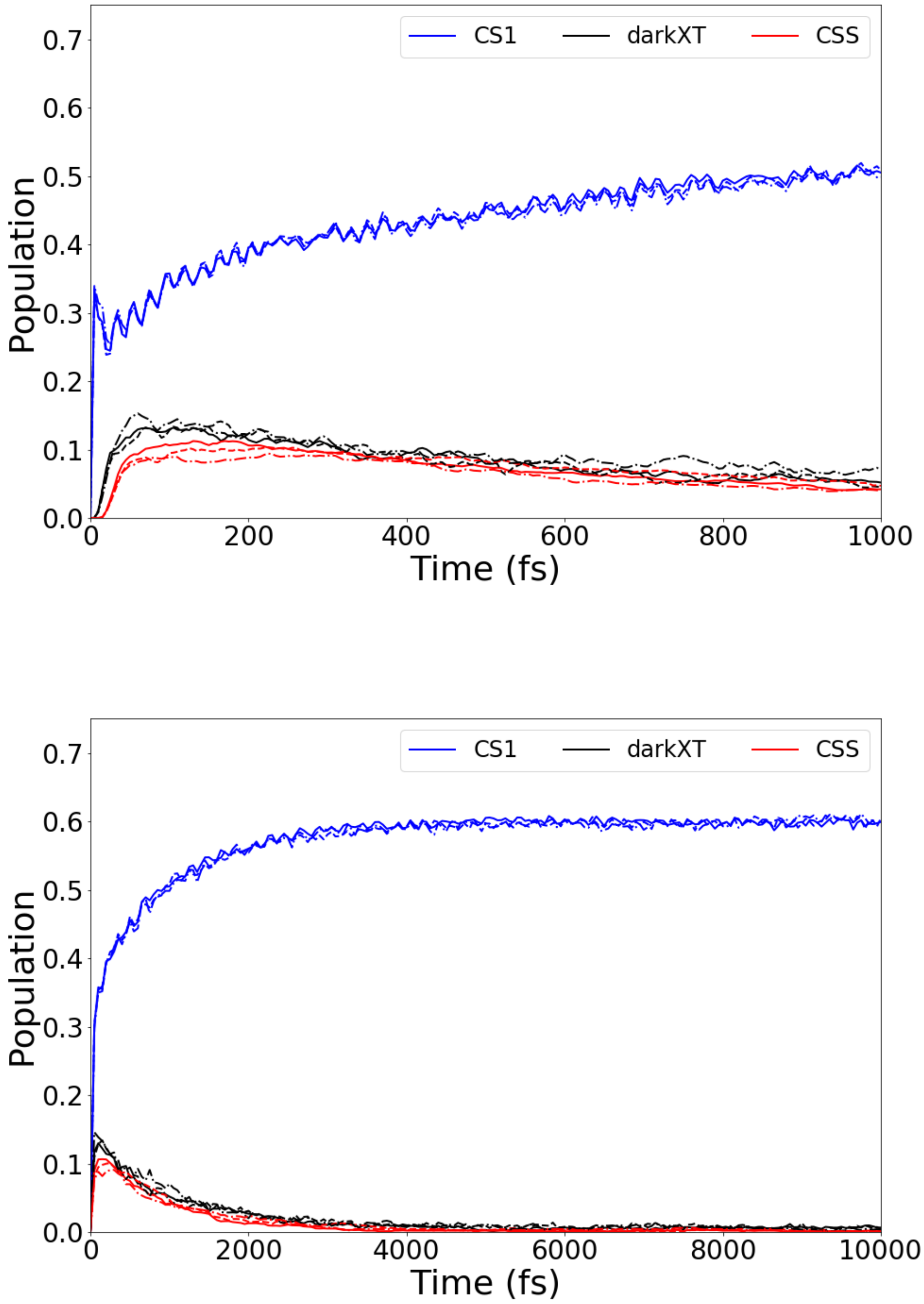

(b)

Figure S3. Convergence of time-dependent populations with respect to integration time step for the fullerene-oligothiophene system, from X-SH. Panels (a) and (b) show the same data on the 1 ps and 10 ps time scales. The solid, dashed, and dash-dotted lines correspond to a nuclear time step of 0.1 fs, 0.05 fs, and 0.01 fs, respectively. The electronic time step is one-fifth of the nuclear time step in each case, 0.02 fs, 0.01 fs, and 0.002 fs, respectively.

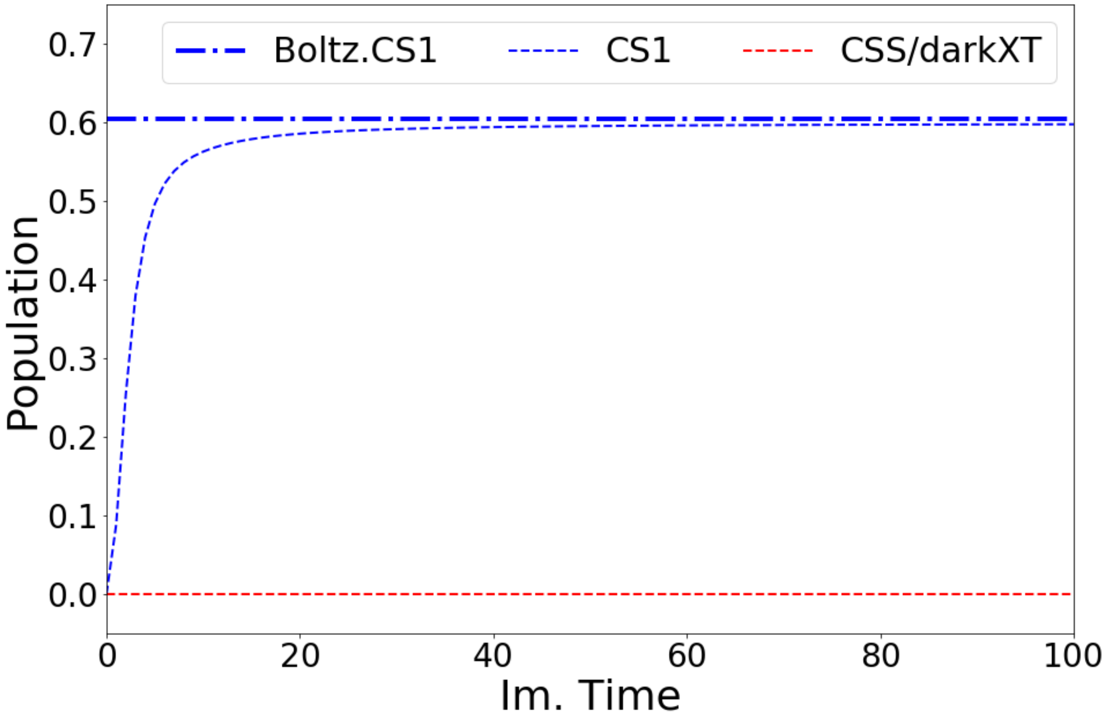

Figure S4. The population of electronic states from imaginary time propagation (dash lines). The quantum equilibrium population (the converged value of the simulation) of CS1 is close to the thermal equilibrium value at 300 K (Boltz. CS1; dash-dotted line). In addition, both darkXT and CSS have quantum equilibrium populations equal to zero, as expected at 0 K.

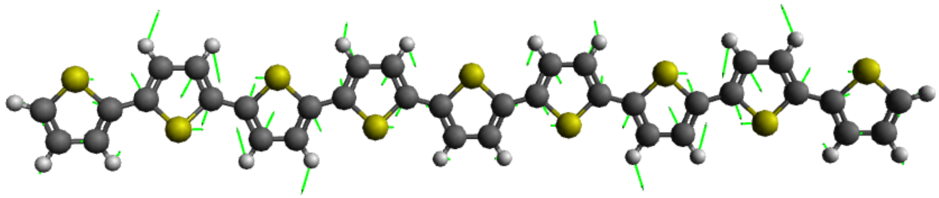

Mode 83: Frequency 734 cm^{-1}

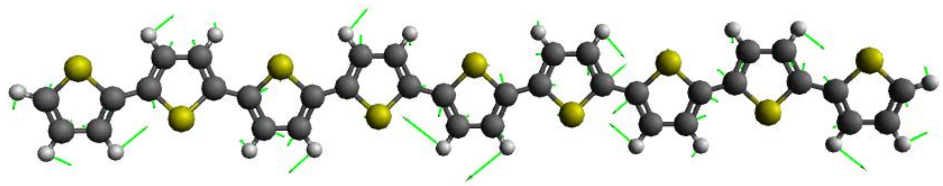

Mode 135: Frequency 1246 cm^{-1}

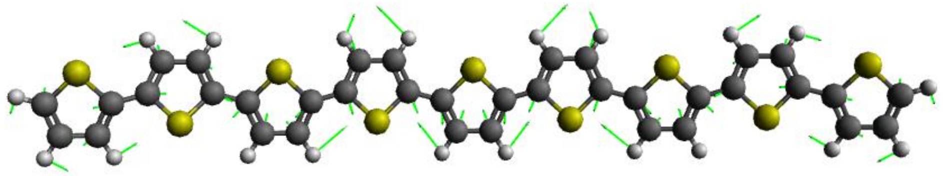

Mode 141: Frequency 1279 cm^{-1}

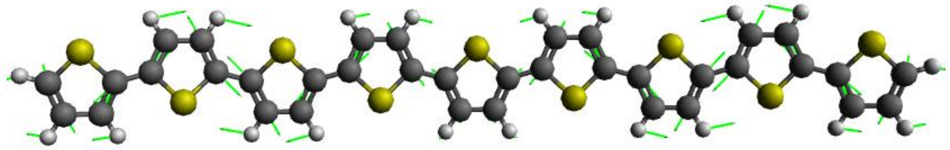

Mode 168: Frequency 1616 cm^{-1}

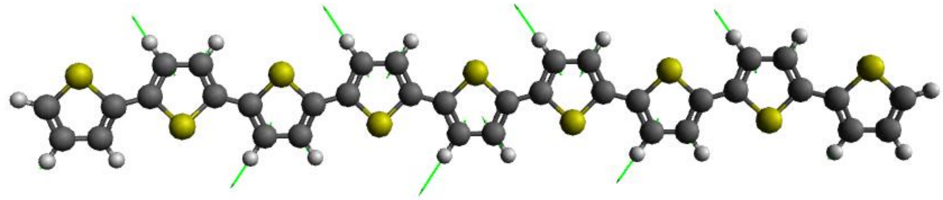

Mode 170: Frequency 3213 cm^{-1}

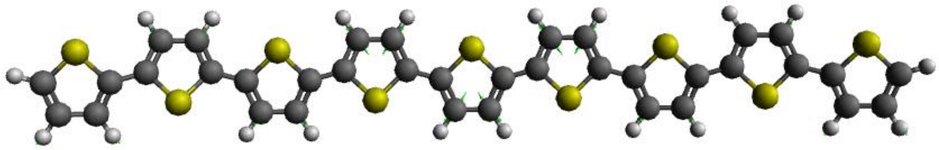

Mode 185: Frequency 3228 cm^{-1}

Figure S5. Six vibrational modes that are active during the dynamics from the normal mode analysis in the X-SH method.

Reference

(1) Huix-Rotllant, M.; Tamura, H.; Burghardt, I., Concurrent Effects of Delocalization and Internal Conversion Tune Charge Separation at Regioregular Polythiophene-Fullerene Heterojunctions. *J. Phys. Chem. Lett.* **2015,** *6*, 1702-1708.
(2) D.A. Case, I. Y. B.-S., S.R. Brozell, D.S. Cerutti, T.E. Cheatham, III, V.W.D. Cruzeiro, T.A. Darden,; R.E. Duke, D. G., M.K. Gilson, H. Gohlke, A.W. Goetz, D. Greene, R Harris, N. Homeyer, Y. Huang,; S. Izadi, A. K., T. Kurtzman, T.S. Lee, S. LeGrand, P. Li, C. Lin, J. Liu, T. Luchko, R. Luo, D.J.; Mermelstein, K. M. M., Y. Miao, G. Monard, C. Nguyen, H. Nguyen, I. Omelyan, A. Onufriev, F. Pan, R.; Qi, D. R. R., A. Roitberg, C. Sagui, S. Schott-Verdugo, J. Shen, C.L. Simmerling, J. Smith, R. SalomonFerrer, J. Swails, R.C. Walker, J. Wang, H. Wei, R.M. Wolf, X. Wu, L. Xiao, D.M. York and P.A. Kollman, *AMBER 2018, University of California, San Francisco* **2018**.
(3) Yanai, T.; Tew, D. P.; Handy, N. C., A New Hybrid Exchange-Correlation Functional Using the Coulomb-Attenuating Method (CAM-B3LYP). *Chem. Phys. Lett.* **2004,** *393*, 51-57.
(4) Becke, A. D., Density-Functional Thermochemistry .3. The Role of Exact Exchange. *J. Chem. Phys.* **1993,** *98*, 5648-5652.
(5) Lee, C. T.; Yang, W. T.; Parr, R. G., Development of the Colle-Salvetti Correlation-Energy Formula into a Functional of the Electron-Density. *Phys. Rev. B* **1988,** *37*, 785-789.
(6) Stephens, P. J.; Devlin, F. J.; Chabalowski, C. F.; Frisch, M. J., Ab-Initio Calculation of Vibrational Absorption and Circular-Dichroism Spectra Using Density-Functional Force-Fields. *J. Phys. Chem.* **1994,** *98*, 11623-11627.
(7) Spencer, J.; Gajdos, F.; Blumberger, J., FOB-SH: Fragment Orbital-Based Surface Hopping for Charge Carrier Transport in Organic and Biological Molecules and Materials. *J. Chem. Phys.* **2016,** *145*, 064102.
(8) Shenai, P. M.; Fernandez-Alberti, S.; Bricker, W. P.; Tretiak, S.; Zhao, Y., Internal Conversion and Vibrational Energy Redistribution in Chlorophyll A. *J. Phys. Chem. B* **2016,** *120*, 49-58.
